# Who uses connected health technologies after a cancer diagnosis? evidence from the US Health Information National Trends Survey

**DOI:** 10.1007/s11764-024-01615-1

**Published:** 2024-05-14

**Authors:** Isaiah Gitonga, Deirdre Desmond, Rebecca Maguire

**Affiliations:** 1 Department of Psychology, Maynooth University, Maynooth, Ireland; 2 Assisting Living and Learning Institute, Maynooth University, Maynooth, Ireland

**Keywords:** Cancer, Connected health technologies, Survivorship, Analysis, Health Information National Trends Survey

## Abstract

**Purpose:**

As the number of people living with and beyond cancer increases, connected health technologies offer promise to enhance access to care and support, while reducing costs. However, uptake of connected health technologies may vary depending on sociodemographic and health-related variables. This study aimed to investigate demographic and health predictors of connected health technology use among people living with and beyond cancer.

**Methods:**

Cross-sectional data from the US Health Information National Trends Survey Version 5 Cycle 4 (H5c4) was used. Regression analysis was used to examine associations between sociodemographic factors and the use of connected health technologies. The sample was restricted to individuals who self-reported a cancer diagnosis or history of cancer.

**Results:**

In this cycle, 626 respondents self-reported a cancer diagnosis, with 41.1% using connected health technologies (health and wellness apps and/or wearable devices). Most were female (58.9%) and white (82.5%); 43.4% had graduated college or higher education. One third (33.6%) had a household income of $75,000 or more. Respondents who were younger, have higher education, were living as married, had higher incomes, had higher self-rated health and had higher health-related self-efficacy were significantly more likely to use connected health technologies. There were no significant associations between gender, race, stratum, time since diagnosis, history of anxiety or depression, and use of connected health technologies among people living with and beyond cancer.

**Conclusions:**

Connected health technology use among people living with and beyond cancer is associated with sociodemographic factors. Future research should examine these demographic disparities as the use of connected health technologies in healthcare continues to gather momentum.

**Implications for cancer survivors:**

The study underscores a disparity in connected heath technology usage among people living with and beyond cancer. There is a pressing need for research into adoption barriers and interventions to ensure equitable digital healthcare integration among this population, especially with the heightened adoption of technology post COVID-19 pandemic.

## Introduction

The number of people living with and beyond cancer (PLWBC) continues to increase globally due to improvements in early detection and treatment and the aging world population [1]. Cancer is now considered a long-term illness with roughly half of cancer patients surviving a period of 10 or more years [2]. Despite the improved cancer survival rates PLWBC have diverse unmet needs [3]. Many PLWBC experience chronic physical symptoms from treatment side effects [4, 5], psychological distress, fatigue, sleep problems, and decreased quality of life [6] and could benefit from ongoing care and support [7, 8].

The growing population of PLWBC requires expansion of healthcare services to meet their needs and to reduce access gaps [9, 10]. As technology continues to be integrated into both healthcare and society at large, digital technologies offer promising possibilities for organization and delivery of care and support [11]. Connected health technologies (CHT), an umbrella term that encompasses concepts of e-health, mhealth, telehealth, and wearables, among other technologies, differ from other technologies in that they offer a two-way flow of information that involves gathering, analysis and return of user data to provide insights to patients and health care professionals [12, 13]. CHT may be effective in supporting patient education, self-management and personalized support in PLWBC [14–16]. Additionally, CHT could reduce logistical barriers associated with in-person care and participation such as time and travel burden [11], making them especially useful in the cancer survivorship context [17, 18].

Adoption and usage of CHT have been widely examined across various patient populations [19, 20], with a growing body of research in the aftermath of the global COVID-19 pandemic [21–23]. These studies report mixed but promising findings on CHT uptake and efficiency [18, 23]. Studies conducted among people with cancer have reported variations in uptake and efficacy [17], indicating the need for further studies to examine predictors, preferences, and attitudes towards use and adoption. Moreover, a recent study examining technology usage over the course of a decade found that while the prevalence and adoption of digital health among cancer PLWBC has continued to rise, the digital divide remains prevalent in this population [24, 25]. The digital divide has been associated with health inequities, as differential access to or usage of CHT across various demographics may result in uneven health outcomes [24]. A recent review by Yao et al. (2022) found that CHT can foster health disparities based on individuals’ ability to adopt technology and their health outcomes, influenced by factors like age, race, location, economic status, education, health status, and eHealth literacy [26]. In their analysis of health disparities, Saeed and Masters (2021) posited that while technological advancements in healthcare aim to enhance outcomes, it is imperative to ensure equitable distribution of these outcomes across various demographics [27]. Moreover, the World Health Organization’s digital health strategy 2020–2025 underlines the importance of acknowledging and addressing demographic disparities when implementing CHT to ensure equitable health outcomes [28].

Despite significant evidence linking socioeconomic factors, access to technology, and digital literacy to technology usage and adoption [20, 29], there is limited research on these associations amidst the rapidly evolving technology landscape. In this study, we used the Health Information National Trends Survey (HINTS) Five, Cycle 4 data [30, 31] to investigate demographic and health-related variables associated with CHT use among PLWBC. The National Cancer Institute (NCI) has conducted HINTS every few years since 2003 to assess health-related information use among civilian, non-institutionalized adults aged 18 or older in the US. HINTS provides the NCI with a comprehensive assessment of the American public’s access to and use of information about cancer across the cancer care continuum from cancer prevention, early detection, diagnosis, treatment, and survivorship.

## Materials and methods

### Study design and procedures

This study used the 5th version of HINTS dataset (HINTS 5 Cycle 4; H5c4) which was collected solely by mail between February 2020 and June 2020. As with prior HINTS iterations, the sampling frame for Cycle 4 consisted of a database of addresses used by Marketing Systems Group to provide random samples of all non-vacant residential addresses in the United States. A two-stage stratified random sampling methodology was used. In the first stage, residential addresses across the United States were selected. In the second stage, one adult was selected within each sampled household. The full sampling and weighting process of H5c4 is described in the HINTS methodology report [31].

### Participants

The total number of addresses selected for Cycle 4 was 15,350. Eligible data was obtained from 3865 respondents, of which 16.2% (*n* = 626) were PLWBC (i.e., respondents who answered “Yes” to the survey item “*have you ever been diagnosed with cancer?*”) [32]. Respondents who responded “no” to this question and those who did not respond to the question (or whose data were missing) were excluded from these analyses.

### Measures

#### Sociodemographic information and disease history

Data were gathered on participants’ age, sex at birth, marital status, the highest level of education, household annual income, race/ethnicity, region/stratum, and years since diagnosis.

#### Use of connected health technologies

Use of connected health technologies (health or wellness apps and wearable devices) was determined based on an affirmative response to either of the following two questions “*In the past 12 months, have you used any of these health or wellness apps?*” and “*In the last 12 months, have you used an electronic wearable device to monitor or track your health or activity? For example, a Fitbit, AppleWatch or Garmin Vivofit…*” The first question was preceded by a question that required respondents to indicate if they had any “apps” related to health and wellness. We did not analyze this question as our focus was on active use of the “apps.”

#### Self-rated health

Self-rated health was measured using a single item on a five-point Likert scale (from 1 = excellent to 5 = poor). For purposes of analysis, we dichotomized responses by combining “*excellent, very good and good*” into one category and “*fair and poo*r” into another category; combined categories were renamed “*good*” and “*poor*,” respectively.

#### Health-related self-efficacy

Health-related self-efficacy was measured using a single item asking respondents how confident they were in their ability to take good care of their health, on a five-point Likert scale (1 = *completely confident* to 5 = *not confident at all*). As with the self-rated health measure, for purposes of analysis, we reduced the five categories into a binary variable (confident/not confident) by combining “*completely confident, very confident*, *somewhat confident*” into one category and “*a little confident and not confident at all*” into another category.

#### History of depression or anxiety

Lifetime history of depression or anxiety was measured by the item “Has *a doctor or other health professional ever told you that you had depression or anxiety disorder?*” *(yes/no)*.

### Data analysis

The Statistical Package for Social Sciences (SPSS) was used for analysis. We restricted our present analysis to respondents who self-reported a cancer diagnosis. Descriptive statistics were used to examine the usage of CHT (health apps and wearable devices). Chi-squared tests and multivariable regression were conducted to assess associations between use of CHT and sociodemographic characteristics, and general health status, including the self-reported history of psychological disorders (anxiety and/or depression), self-rated health, and health related self-efficacy. Finally, multivariate logistic regression was conducted to identify predictors of CH usage. Statistical significance was set at *p* < 0.05.

## Results

### Sample characteristics

The sample characteristics are provided in Table 1. A total of 626 respondents reported to have ever been diagnosed with cancer. The majority were female (58.9%) and over the age of 65 (63.6%). Nearly half of the respondents were married (49%). Over two-thirds had completed at least some college education (71%), and approximately half had an annual household income above $50,000 (50.8%). Most identified as white (82.5%) and lived in high minority areas (57.2%). 48.4% were diagnosed with cancer 11 or more years ago. Almost three quarters of the PLWBC (74%) rated their general health as “good” and 69% “*felt confident*” in their ability to take care of their health. About a quarter of the respondents (24.4%) had been diagnosed with depression or anxiety.

**Table 1:** Sociodemographic characteristics and disease history of people living with and beyond cancer

Variable	Category	Frequency (*N* = 626)	Percentage (%)
Age in years	18–64 years	225	36.4
	65 and above	393	63.6
	*Missing/non-response*	8	
Sex at birth	Male	256	41.1
	Female	367	58.9
	*Missing/non-response*	3	
Marital status	Married	298	49.0
	Living as married	16	2.6
	Divorced	105	17.3
	Widowed	106	17.4
	Separated	10	1.6
	Single, never been married	73	12.0
	*Missing/non-response*	18	
Level of education	Less than high school	43	7.1
	High school graduate	132	21.9
	Some college	167	27.6
	College graduate or more	262	43.4
	*Missing/non-response*	22	
Household income	Less than $20,000	104	19.0
	$20,000 to < $35,000	79	14.4
	$35,000 to < $50,000	87	15.9
	$50,000 to < $75,000	94	17.2
	$75,000 or more	184	33.6
	*Missing/non-response*	78	
Race	White	495	82.5
	Other	105	17.5
	*Missing/non-response*	26	
Stratum	High minority areas	358	57.2
	Low minority areas	268	42.8
Time since diagnosis	Less than 1 year	78	13.3
	2–5 years	110	18.7
	6–10 years	115	19.6
	11 + years	284	48.4
	*Missing/non-response*	39	
Self-rated general health	Poor	162	26.0
	Good	460	74.0
	*Missing/non-response*	4	
Self-rated ability to take good care of their health	Not confident	193	31.0
	Confident	429	69.0
	*Missing/non-response*	4	
Ever been diagnosed of Depression/Anxiety	Yes	151	24.4
	No	468	75.6
	*Missing/non-response*	7	

### Use of connected health technologies

Overall, 41.1% (*n* = 257) of the sample reported using health or wellness apps and/or wearable devices to manage the health and wellbeing in the past year (Table 2).

**Table 2:** Use of connected health technologies among people living with and beyond cancer

Use of CH technology	Frequency (*N* = 626)	Percentage (%)	95% confidence interval	
			Lower	Upper
Use of health wellness apps in the last 12 months	224	35.8	32.1	39.9
Use of wearable devices to track health in the last 12 months	127	20.3	17.4	23.8
Use of connected health technology	257	41.1	37.2	45.2

### Association between demographic variables and CHT use

Table 3 shows associations between sociodemographic variables and CHT usage in the past year. Age, marital status, level of education, and household income were significantly associated with technology use at the bivariate level. PLWBC aged 18–64 years were more likely to use technology compared to those aged 65 and above (OR = 2.65, 95% CI = 1.89–3.72, *p* < 0.001). Those with a college degree or higher had higher odds of technology use compared to those with less than a high school education (OR = 10.69, 95% CI = 3.71–30.76, *p* < 0.001). Similarly, PLWBC with a household income of $75,000 or more had higher odds of technology use compared to those with an income of less than $20,000 (OR = 4.98, 95% CI = 2.93–8.47, *p* < 0.001). Additionally, PLWBC who rated their health as “good” were more likely to use CHT (OR = 2.39, 95% CI = 1.61–3.54, *p* < 0.001) compared to those who rated their health as “poor.” Similarly, respondents who reported being confident about their ability to take good care of their health were more likely to use CHT (OR = 1.50, 95% CI = 1.05–2.13, *p* < 0.001) compared to those who were less confident. Sex at birth, race, time since diagnosis, and lifetime diagnosis of depression/anxiety were not significantly associated with CHT use.

**Table 3:** Factors associated with connected health technologies among PLWBC

Variable	Category	Use of technology [*n* (%)]		OR [95% CI]	Sig. (*p*-value)
		No	Yes		
Age in years	18–64 years	98 (27.1%)	127 (49.6%)	2.65 [1.89; 3.72]	< 0.001
	65 and above	264 (72.9%)	129 (50.4%)	Ref	
Sex at birth	Male	148 (40.4%)	108 (42.0%)	1.07 [0.77; 1.48]	0.692
	Female	218 (59.6%)	149 (58.0%)	Ref	
Marital status	Married	150 (42.3%)	148 (58.5%)	1.59 [0.94; 2.68]	0.084
	Living as married	4 (1.1%)	12 (4.7%)	4.82 [1.41; 16.43]	0.012
	Divorced	68 (19.2%)	37 (14.6%)	0.87 [0.47; 1.62]	0.671
	Widowed	81 (22.8%)	25 (9.9%)	0.50 [0.26; 0.95]	0.035
	Separated	7 (2.0%)	3 (1.2%)	0.69 [0.16; 2.89]	0.610
	Single, never been married	45 (12.7%)	28 (11.1%)	Ref	
Level of education	Less than high school	39 (11.1%)	4 (1.6%)	Ref	
	High school graduate	92 (26.2%)	40 (15.8%)	4.24 [1.42; 12.66]	0.010
	Some college	95 (27.1%)	72 (28.5%)	7.39 [2.53; 21.62]	< 0.001
	College graduate or more	125 (35.6%)	137 (54.2%)	10.69 [3.71; 30.76]	< 0.001
Household income	Less than $20,000	77 (24.6%)	27 (11.5%)	Ref	
	$20,000 to < $35,000	61 (19.5%)	18 (7.7%)	0.84 [0.42; 1.67]	0.621
	$35,000 to < $50,000	59 (18.8%)	28 (11.9%)	1.35 [0.72; 2.54]	0.345
	$50,000 to < $75,000	49 (15.7%)	45 (19.1%)	2.62 [1.44; 4.76]	0.002
	$75,000 or more	67 (21.4%)	117 (49.8%)	4.98 [2.93; 8.47]	< 0.001
Race	White	282 (81.3%)	213 (84.2%)	1.23 [0.80; 1.89]	0.353
	Other	65 (18.7%)	40 (15.8%)	Ref	
Stratum	High minority areas	216 (58.5%)	142 (55.3%)	0.87 [0.63; 1.21]	0.414
	Low minority areas	153 (41.5%)	115 (44.7%)	Ref	
Time since diagnosis	Less than 1 year	43 (12.6%)	35 (14.2%)	1.15 [0.69; 1.90]	0.599
	2–5 years	61 (17.9%)	49 (19.8%)	1.13 [0.72; 1.76]	0.589
	6–10 years	70 (20.6%)	45 (18.2%)	0.90 [0.58; 1.41]	0.656
	11 + years	166 (48.8%)	118 (47.8%)	Ref	
Self-rated general health	Poor	119 (32.5%)	43 (16.8%)	Ref	
	Good	247 (67.5%)	213 (83.2%)	2.39 [161; 3.54]	< 0.001
Self-rated ability to take good care of their health	Not confident	126 (34.5%)	67 (26.1%)	Ref	
	Confident	239 (65.5%)	190 (73.9%)	1.50 [105; 2.13]	0.025
Lifetime diagnosis of depression/anxiety	Yes	84 (23.2%)	67 (26.1%)	1.17 [0.81; 1.69]	0.414
	No	278 (76.8%)	190 (73.9%)	Ref	

###  CHT use among PLWBC

In adjusted models, age, marital status, household income, and self-rated general health were significant, independent predictors of CHT usage. Specifically, those aged 18–64 years were significant more likely to use CHT (aOR = 2.06, 95% CI = 1.35–3.13, *p* = 0.001) than older adults. PLWBC with an income of $75,000 were more likely to use CHT (aOR = 3.10, 95% CI = 1.48–6.49, *p* = 0.003) compared to those with less than $20,000. Those who rated their general health as good were more likely to use CHT (aOR = 1.92, 95% CI = 1.09–3.38, *p* = 0.023) compared to those who rated their health as poor (see Table 4).

**Table 4:** Sociodemographic and health related predictors of connected health technologies among PLWBC

Variable	Category	aOR [95% CI]	Sig
Age in years	18–64 years	2.06 [1.35; 3.13]	0.001
	65 and above	Ref	
Marital status	Married	1.09 [0.56; 2.14]	0.804
	Living as married	7.66 [1.37; 42.63]	0.020
	Divorced	0.80 [0.39; 1.65]	0.543
	Widowed	1.15 [0.52; 2.56]	0.732
	Separated	2.26 [0.45; 11.28]	0.319
	Single, never been married	Ref	
Level of education	Less than high school	Ref	
	High school graduate	2.49 [0.65; 9.60]	0.185
	Some college	3.35 [0.88; 12.68]	0.075
	College graduate or more	2.65 [0.69; 10.17]	0.155
Household income	Less than $20,000	Ref	
	$20,000 to < $35,000	1.06 [0.47; 2.35]	0.895
	$35,000 to < $50,000	1.09 [0.52; 2.30]	0.819
	$50,000 to < $75,000	1.86 [0.89; 3.90]	0.101
	$75,000 or more	3.10 [1.48; 6.49]	0.003
Self-rated general health	Poor	Ref	
	Good	1.92 [1.09; 3.38]	0.023
Self-rated ability to take good care of their health	Not confident	Ref	
	Confident	0.89 [0.54; 1.47]	0.661

## Discussion

This study investigated usage and sociodemographic predictors of a subset of CHT (health apps and wearable devices) among PLWBC using a nationally representative sample of United States adults. We found that nearly half of PLWBC used some the form of CHT; however, usage varied across sociodemographic and health-related variables. Our study found higher CHT usage in PLWBC compared to analysis of previous HINTS datasets [33, 34], suggesting that usage is increasing among this population. This underscores earlier findings on the potential of CHT, particularly emerging technologies such as health and wellness apps and wearable devices [15, 33], to offer new avenues for reaching PLWBC with targeted interventions.

Our findings suggest that CHT usage varies across various sociodemographic and health related variables. We found that younger individuals, with higher levels of education, higher income, and living as married were more likely to use CHT. These findings are consistent with prior studies which reported disparities in the use of CHTs in PLWBC [24]. This suggests that, although CHT usage and adoption is on the rise, a digital divide still persists and this could potentially worsen health disparities [27], implying that certain individuals may be “digitally disconnected” and therefore unable to use CHT for their heath needs [35, 36]. Furthermore, it is important to address these disparities in CHT usage to prevent exacerbation of existing health inequalities as was the case during the COVID-19 pandemic [37]. This may include working to improve access to and knowledge of these technologies among PLWBC with lower socioeconomic status, as well as ensuring that these technologies are designed in a way that is accessible and usable for all people impacted by cancer, whose population is on the rise, regardless of their socioeconomic status.

The finding that there was no association between a history of anxiety and depression and usage of CHT suggests that mental health history may not play a significant role in an individual's likelihood of using CHT. Although there is evidence suggesting that severe mental illness could be a barrier to technology usage [38], our findings are consistent with a previous study that found people with a history of depression and anxiety used CHT at similar rates to the general population.[39]. This suggests that mental health history may not be a barrier to CHT use for managing conditions such as cancer. Furthermore, it suggests that CHT may be a useful tool for individuals with a history of mental health conditions who are managing other health issues. However, it is also possible that the questions used in this survey were not sensitive enough to establish this association, and further research is needed to examine the association.

Our finding that PLWBC with higher self-rated health were more likely to use CHT is consistent with previous research [40, 41]. This suggests that technologies designed to enhance self-rated health and self-efficacy may be particularly effective in engaging PLWBC and promoting their use of these technologies. For instance, by providing features that enable individuals to track and manage their health, set goals, and receive feedback, these technologies may help foster a sense of control and confidence in managing their health. However, further research is needed to better understand the mechanisms through which these factors influence technology adoption and usage among PLWBC and to identify strategies for promoting the adoption and effective use.

## Study limitations and strengths

There are several limitations to this study. Firstly, the cross-sectional nature of the data means that it can only provide information on associations of CHT usage rather than causal relationships. Secondly, there is the possibility of recall bias since the survey relied on self-reported information and required participants to recall past behaviors, such as their use of CHT in the past year. Third, the HINTS only examined a subset of CHT (health apps and wearable devices), and this is not inclusive of all CHT such as electronic health records, whose usage might be different. Additionally, due to the nature of questions used in the survey, we were unable to obtain specific information on the types, content, design, and characteristics of health apps, their frequency of use, number of apps, and costs. Finally, data collection in this HINTS cycle was impacted by COVID-19 mitigations which reduced the workforce available for distributing survey packets, leading to a modified mailing schedule with longer intervals between mailings and possible delays [31]. A strength of this study is that the responses in this iteration (H5c4) were collected through mail, thereby avoiding any bias that may arise from “using technology to study technology.” Secondly, the sample used for the study was nationally representative, enhancing the generalizability of the findings to the broader population.

## Conclusion

While usage of CHT among PLWBC is on the rise, this study has shown that use varies across sociodemographic and health-related variables, with those who are older and with lower SES less likely to use CHT. To ensure that the expansion of CHT does not worsen existing healthcare disparities, future research should focus on addressing the barriers to usage and adoption and expanding their reach to all subgroups of PLWBC. Additionally, future studies should examine barriers and enablers to use of these technologies, considering the increasing digitalization of healthcare, particularly in the aftermath of COVID 19 pandemic. Finally, as more recent, and richer iterations of HINTS datasets become available, future analyses should include a broader spectrum of connected health technologies.

## Data Availability

This data is publicly available and downloadable from the HINTS website [31]. The data that support the findings for this study are openly available in HINTS Public Use Dataset available at https://hints.cancer.gov/data/download-data.aspx.
